# Disentangling evolutionary, geometric and ecological components of the elevational gradient of diversity

**DOI:** 10.1093/evlett/qrae048

**Published:** 2024-09-22

**Authors:** Leonel Herrera-Alsina, Rossina Parvanova, Jacinta Guirguis, Greta Bocedi, Liam Trethowan, Lesley T Lancaster, Justin M J Travis

**Affiliations:** School of Biological Sciences, University of Aberdeen, Aberdeen, United Kingdom; School of Biological Sciences, University of Aberdeen, Aberdeen, United Kingdom; MacroBiodiversity Lab, School of Biological Sciences, Queen’s University Belfast, Belfast, United Kingdom; School of Biological Sciences, University of Aberdeen, Aberdeen, United Kingdom; Royal Botanic Gardens Kew, London, United Kingdom; School of Biological Sciences, University of Aberdeen, Aberdeen, United Kingdom; School of Biological Sciences, University of Aberdeen, Aberdeen, United Kingdom

**Keywords:** diversification rate, lowland diversity, endemic clade, local adaptation, area-dependent process

## Abstract

Despite the high importance and risk of mountain ecosystems in global biodiversity conservation, the mechanisms giving rise to and maintaining elevational biodiversity gradients are poorly understood, limiting predictions of future responses. Species richness peaks at lowlands for many taxa, which might be a consequence of mountain shape, reducing available area in highlands. For other taxa, diversity can be highest at mid elevations, suggesting the presence of mechanisms that counteract the influence of geometry. Here, we mechanistically investigate the role of mountain geometry (smaller at the peak) interaction with ecological niche width, diversification, and altitudinal dispersal to investigate the relative roles of these processes in shaping elevational biodiversity gradients. We simulated landscapes and lineages until species richness stop increasing and showed that the disproportionately large area of lowlands provides opportunity for higher species accumulation than any other elevation, even when available niche width and per-capita diversification rate are uniform across altitudes. Regardless of the underlying Elevational Diversity Gradient, altitudinal dispersal always plays a stronger role in maintaining highland than lowland diversity, due to unequal areas involved. To empirically test these predictions resulting from our model, we fit dynamic models of diversification and altitudinal dispersal to three mountainous endemic radiations whose species richness peaks in mid and high-elevation. We find that highland diversity is explained by increased diversification rates with elevation in Fijian bees, whereas niche availability is more likely to explain high altitude diversity in frailejon bushes and earless frogs, suggesting these clades are still growing. Our model and findings provide a new framework for distinguishing drivers of diversity dynamics on mountainsides and allow to detect the presence of clade-specific mechanisms underlying the geometry-diversity relationship. Understanding of these ecological and evolutionary forces can allow increased predictability of how ongoing land use and climate changes will impact future highland biodiversity.

## Introduction

Mountains hold many endemic groups (Jetz et al., 2004) and a large proportion of species richness (Orme et al., 2005; Rahbek et al., 2019), making their study and conservation vital for global biodiversity. In particular, understanding the ecological and evolutionary dynamics linked to mountain systems is paramount in the current human-induced climate change. At present, montane biodiversity face rapidly changing biotic and abiotic conditions, largely driven by human-induced climate change. For instance, climate change is characterized by shifts in species elevational ranges (Comte et al., 2024). As temperatures rise, species follow their temperature niche towards higher elevations until there is nowhere to go (Vitasse et al., 2021) or are negatively affected by new competitors migrating from lower elevations (Alexander et al., 2015). Because available area inherently varies with elevation (e.g., a conic-shaped mountain; Elsen & Tingley, 2015), any area-dependent ecological or evolutionary process will be altered. Key processes such as local adaptation, net diversification (i.e., speciation minus extinction), and ecological niche width are all area-dependent and, thus, should be analyzed together with mountain geometry to detect and predict global changes in biodiversity.

The uneven distribution of species across elevational gradients can result from different rates of diversification. Net diversification rates can peak at high elevations (C. E. Hughes & Atchison, 2015; Ebersbach et al., 2017; Esquerré et al., 2019). Landscape ruggedness (Guegan et al., 1998; Ohlemüller et al., 2008) and temporal variation in connectivity (Flantua et al., 2019) increase chances of species divergence at high altitude, which is reflected in, for instance, the large number of montane young endemics (Fjeldså, 1994). Likewise, increased topographic complexity in high-elevation regions may increase the community niche width via fine scale habitat divergence (Henriques et al., 2022; Malpica et al., 2017). However, from a geographic perspective, high elevations may increase extinction rates due to limited habitat area and dispersal limitation (“sky island” effect). Increased area at low elevation means that species can attain large range sizes which increases chances for speciation and simultaneously reduces extinction probability (Gaston, 1998). From an ecological perspective, lowlands can be cradles of species origination via intense ecological interactions (Pigot et al., 2016) whereas adaptation to cold highlands might constrain further adaptation (Pincheira-Donoso et al., 2013) to changing climatic conditions, increasing extinction rates (Sinervo et al., 2010). In contrast, high-elevation areas tend to exhibit slower velocities of climate change in comparison to lowlands (Loarie et al., 2009) and often invoke rain shadow effects that shield species from periods of desertification and drought. These factors thus also limit extinction rates in highland compared to lowland clades (Lancaster & Kay, 2013). In summary, how elevation affects diversification rates is unclear.

At what elevation does species richness peak? Locally, the number of species able to co-exist within a set area (here termed the community niche width or local saturation) is likely to vary with elevation. Higher productivity and longer growing seasons are expected at lower elevations which amounts to greater ecological niche width (Price et al., 2014). When considering the species-area relationship, lowlands are expected to be richer than highlands because their larger area facilitates species accumulation (Polato et al., 2018). That local diversity is often highest in low elevational bands supports a decrease in niche width with elevation. In fact, in a “lowlands-have-it-all” scenario, lowlands could have both wide available niche width and high diversification rates. Empirical data shows that local species richness decreases with elevation Elevational Diversity Gradient (EDG), but other elevation-richness relationships are possible (Table 1). That diversity can be the highest at elevations other than lowlands suggests the presence of mechanisms in place that counteract the influence of geometry in mountain systems.

**Table 1.** Overview of 50 studies on diversity distribution across elevation. A list of references for this table is found in Supplementary File 2. A more detailed version of this table can be found in Supplementary File 1.

Taxa	Species richness pattern	Pattern cont.	Location	Mountain chain	Significant/driving factors	Source
Birds	Low-elevation peak	Mid-elevation sharp decline (1,250 m), high-elevation plateau	Bolivia	Andes (3,400 m)	Elevation/MDE; Elevation/area; Regional Species pool	Herzog et al. (2005)
Birds	Mid-elevation asymmetrical peak	Eastern slope—Mid-elevation asymmetrical hump- at 1,200–1,700 m Western slope—Mid-elevation asymmetrical hump- at 2,200 m	Horn of Africa (Ethiopia, Eritrea, Djibouti, Somalia)	Eastern and western highlands (4,200 m); dry side and wet side (3,500 and 4,200)	Temperature range, Precipitation, Productivity	Abebe et al. (2019)
Birds	Monotonic decrease		Papua New Guinea	Mt Wilhelm (4,509 m)	Habitat complexity (esp. Insectivores). Frugivores—Available surface area. Temperature—Total, insectivore‐nectarivores, pure nectarivores	Sam et al. (2018)
Birds	Low-elevation peak	Endemics—mid-elevation peak (2,200–2,800); Larger range species → high-elevation peak Small range species → low elevation peak Passerines peak at slightly higher elevations	China	Hengduan Mountains (6,000 m)	Temperature, Normalized difference vegetation index, Enhanced vegetation index, Seasonality, Large-ranged species, and endemics—MDE	Wu et al. (2013)
Birds	Monotonic decrease & Mid-elevation peak	Monotonic decrease—Slopes with a complete elevational gradient Mid-elevation peak—Internal slopes with truncated lower elevational belts Mid-elevation peak of tropical Andean species	Colombia	Andean Cordilleras; La Macarena; Santa Marta	Area of the altitudinal belts	Kattan and Franco (2004)
Birds	Mid-elevation Hump		India	Himalaya, Sikkim (4,500 m)	Actual evapotranspiration (primary productivity); Plant species richness; Shrub density; Basal area of trees	Acharya et al. (2011)
Reptiles and Amphibians	Low-elevation peak and Mid-elevation peak		Kenya	Mt. Kenya (5,200 m)	Rainfall; Temperature	Malonza (2015)
Amphibians	Monotonic decrease	Elevation specialists with narrow ranges predominate	Nepal	Himalaya, Koshi Basin (3,430 m)	Elevation; Surface area; Humidity	Khatiwada et al. (2019)
Amphibians (Frogs)	Mid-elevation Hump	Non-endemics—Monotonic, lower than total peak Endemics—higher than total peak	China	Hengduan Mountains	Total trend—Temperature; Precipitation; Spp. Range Non-endemics—Water and Energy Endemics—MDE	Fu et al. (2006)
Amphibians	Mid-elevation Hump	Endemics—higher elevational peak (2,000–2,500 m)	India	Himalaya, Sikkim (300–4,600 m)	Endemics and Large-range species—Mid-domain effect; Mean annual precipitation; Non-endemics and small-range species—Actual evapotranspiration	Chettri and Acharya (2020)
Non-volant small mammals	Mid-elevation Hump		Costa Rica	Caribbean slope of the Río Penas Blancas	Mid-domain model	McCain (2004)
Non-volant small mammals	Mid-elevation Hump	Boundary between low and high-elevation assemblages falls between 1,200 m and 1,700 m or between 1,700 and 1,800 m	Malaysia, Borneo	Mount Kinabalu (4,101 m)	Highland and a lowland assemblage overlapped, Plant taxa diversity maximum, rainfall and humidity reached maxima	Nor (2008)
Rodents, insectivores, lagomorphs	Asymmetric Mid-elevation Hump	Endemics—hump shape—two peaks at 3,150 and 3,300–4,300 m Non-endemics—hump-shaped peak at lower elevations (2,400 m). Small range spp.—Hump-peak at 2,800–3,100 m Large-range spp.—hump-peak at 3,750 m	China	Gyirong Valley, Mount Qomolangma, Southern Himalayas (1,800–5,400 m)	Large-ranged species—MDE Endemics—Habitat heterogeneity Water–energy dynamics—total, especially non-endemic species.	Hu et al. (2017)
Rodents, insectivores, lagomorphs	Mid-elevation Hump	Declines at both lower and higher elevations. Elevational distributions of individual species varied considerably.	USA, Utah	Great Basin mountains, Uinta Mountains (4,117 m)	Area (positive) Negative correlation with isolation—broad-elevation species	Rickart (2001)
Insectivores	Mid-elevation hump	Rodents—two peaks—larger peak at lower elevation (1,600–2,000 m); smaller peak at higher elevation (2,800–3,200 m). Endemics—peak at mid-elevation (2,800 m) Non-endemics—two peaks—larger peak at lower elevation (1,600–2,000 m); smaller peak at 3,200 m.	China	Hengduan Mountains, Gongga Mountain (6,400 m)	Mid-domain effect Rodents—Plant-species richness Non-endemics—Temperature	Wu et al. (2012)
Rodents	Low-elevation & Mid-elevation peak					
Rodents	Mid-elevation Hump	Peak—low elevations tropical semideciduous forest Endemic species—restricted to high-elevation habitats	Mexico	Sierra Mazateca (640–2,600 m); Sierra Mixteca (700–3,000 m)	Increasing: habitat diversity; rainfall; productivity; resource diversity; areas with high rates of speciation	Sánchez-Cordero (2001)
Rodents	Monotonic decrease & Mid-elevation hump	Monotonic decrease—South slope Mid-elevation Hump—Northern slope	China	Qinling Mountains; Mt. Taibai 3,767 m	South slope—Temperature; North slope—mid-domain effect (MDE); Connectivity Larger-ranged species on northern slope—Area and MDE	Shuai et al. (2017)
Insectivores and Rodents	Mid-elevation Hump	300 m—curvilinear, peaking near the area where the transition occurs from montane to mossy forest. 2,000 m—Only the increase phase of the curve is clear.	Philippines	Mount Isarog (2,000 m); Mount Pangasugan (1,150 m), Mount Kitanglad (2,900 m)	Vegetation, Rainfall, Total community abundance, Food resource diversity, Reduced competition, High speciation areas, Habitat diversity	Heaney (2001)
Bats	Monotonic decline		Philippines			
Bats	Mid-elevation Hump	Abrupt decrease at higher elevations	Mexico	Sierra Mazateca (640–2,600 m)	Increasing: habitat diversity; rainfall; productivity; resource diversity; Higher effect of rainfall in Sierra Mazateca	Sánchez-Cordero (2001)
	Low-elevation peak			Sierra Mixteca (700–3,000 m)	Increasing habitat diversity; rainfall; productivity; resource diversity;	Sánchez-Cordero (2001)
Large mammalian Herbivores	Low-elevation unimodal peak	Total—low elevation unimodal peak	Brazil	Itatiaia Massif (2,878 m)	Total—Net primary productivity (main); Temperature Large herbivores—Net primary productivity; Temperature Large omnivores—Temperature Large predators—no significant factor	Lasmar (2021)
Large mammalian Omnivores						
Large mammalian Predators	Non-significant trend					
Fish	Monotonic decrease	Total and non-endemic—Monotonic decrease Endemics—Mid-elevations peak (700–1,500 m)	India	Himalaya-Teesta River (3,800 m)	Water discharge (main) Temperature Basin area (km2)	Bhatt, Manish, and Pandit (2013)
Butterflies	Monotonic decline with low elevation peak		India	Himalaya, Sikkim (4,700)	Actual evapotranspiration; Mean Annual Temperature Tree species richness Shrub species richness Moisture	Acharya and Vijayan (2015)
Moths	Monotonically increasing	Diversity of Larentiinae was highest above 1,800 m.	Ecuador	Andes (2,677 m)	Area; Diversity of potential host-plants; Relatively low predation pressure	Brehm, Sussenbach, and Fiedler (2003)
Steninae Beetles	Monotonic decrease, low elevational peak	Litter-inhabiting and waterfall-associated spp show a mid-elevational peak (1,500 m and 1,000 m respectively). Linear decline for vegetation dwelling species.	Thailand	Doi Inthanon and Doi Pha Hom Pok —2,500 m	Habitat niche width; Area available; Temperature; Mid-domain effect for specific groups.	Betz, Srisuka, and Puthz (2020)
Ants (Formicidae)	Mid-elevation Hump	Each subfamily peaked at mid-elevations	Western USA	4,400 m	Area; geometric constraints (MDE)	Sanders et al. (2002)
Ants (Formicidae)	Mid-elevation Hump	500 and 1,000 m—Indo-Malayan elements dominate Above 2,000 m—Palearctic elements dominate Endemics—High-elevation peak	India	Jammu-Kashmir Himalaya (4,000 m)	Temperature	Bharti et al. (2013)
Tachinids (Diptera)	Monotonic decrease, low elevational peak		North Italy	Alps 2,200 m	Temperature (positive)	Corcos et al. (2017)
Sphecids (Hymenoptera)	Monotonic decrease, low elevational peak				Temperature (positive)	
Hoverflies (Diptera)	Monotonic increase				Temperature (negative)	
Ground beetles (Coleoptera)	Non-significant relationship					
Cavity-nesting Bees (Apidae, Colletidae, Megachilidae)	Monotonic decline	Parasitism rate decreased with elevation across all host groups. Increasingly humped-shaped elevational distribution as the trophic level increased	Tanzania	Mt. Kilimanjaro (1,788 m)	Temperature (strongest relationship)	Mayr et al. (2019)
Predatory Wasps (Eumeninae, Pompilidae, Sphecidae, Crabronidae)	Mid-elevation Hump					
Arthropod Herbivores	Monotonic decline	Cumulative Low-elevation peak for all arthropods	Brazil	Itatiaia Massif (2,878 m)	Total—Temperature (main); Net primary productivity Herbivores—Temperature Omnivores—Temperature Net primary productivity Predators—Net primary productivity	Lasmar (2021)
Arthropod Omnivores	Unimodal decline					
Arthropod Omnivores Predators						
Pterydophytes	Low-elevation Hump	Endemics—Mid-elevation hump at 1,200 m Native—Low-elevation peak at 250 m Alien species—Sharp monotonic decline from 200 m	Italy	Apuan Alps (1,950 m)	Area, habitat heterogeneity	Musciano et al. (2021)
Ptherydophytes	Mid-elevation Hump	Peak found at wettest study site	Bolivia	Andes	Mean annual precipitation; Bryophyte cover on tree branches (proxy for air humidity)	Kessler (2000)
Ptherydophytes	Mid-elevation Hump		Costa Rica	Volcán Barva (2,800 m) & Cerro de la Muerte (2,700–3,400 m).	MDE, temperature, humidity	Kluge, Kessler, and Dunn (2006)
Moss	Mid-elevation Hump		China	Mt. Tuofeng (2,282 m)	MDE (main), habitat complexity, beta diversity	Gao et al. (2021)
Trees (dicot trees, palms, tree ferns)	Low-elevation peak	Lowland diversity peak (300 m)	Costa Rica	Volcan Barva (2,600 m)		Lieberman et al. (1996)
Trees	Low-elevation peak	Monotonic decrease of diversity; high species turnover	Mexico	Cofre de Perote Volcano (4,000)	Temperature	Toledo-Garibaldi and Williams-Linera (2014)
Woody lifeforms—trees and shrubs (Fabaceae, Meliaceae, Annonaceae, Myricaceae, Ericaceae, Monimiaceae, Phyllanthaceae, Pandanaceae, etc.)	Monotonic decrease	Number of families decreasing in species richness was higher than that of families increasing along the elevational gradient	Democratic Republic of the Congo	Kahuzi-Biega National Park, Mitumba Mountains (2,760 m)	N/A	Cirimwami (2019)
Herbacious form (Asteraceae, Aspleniaceae, Dennstaedtiaceae, Lycopodiaceae, Marantaceae, Zingiberaceae)	Monotonic increase	Number of families increasing in species richness was higher than that of families decreasing along the elevational gradient				
Ectomycorrhizal fungi	Mid-elevation Hump	Exception: tomentelloid fungi—monotonal decrease	Borneo	Mount Kinabalu (4,000 m) & Crocker Range (1,800 m)	Environmental factors	Geml et al. (2017)

The number of dispersers and the number of effective altitudinal dispersal (dispersal hereafter) events might also be variable across elevations, influencing the distribution of species within a mountain. The processes involved in successful dispersal involve emigration rates (e.g., which may be higher where population size is larger), facilitation of dispersal (e.g., by wind or freshwater), and availability of niche width for new colonists to settle. Trends in species dispersal across elevations are contentious. While some studies find that lineages are more likely to move to higher elevation (Lenoir et al., 2020; Tingley et al., 2012), other analyses reveal that downhill dispersal events outnumber uphill events and this is responsible for EDG (van Els et al., 2021). Local adaptation to a decreasing temperature with elevation could also influence the rates of dispersal but its relative contribution to EDG has not been described (Lancaster, 2016; Polato et al., 2018). In summary, EDG is thought to be driven by geographic variation in species-level processes of speciation, migration, and extinction, coupled with ecological factors such as habitat area, niche width, and productivity (Cai et al., 2018; Gaston, 2000).

Despite the wealth of research on EDG (Table 1), a comprehensive theory is needed to disentangle: (a) geometry (smaller areas of habitat available towards mountain peaks), (b) diversification asymmetries (faster in highlands or lowlands), (c) niche width (greater in highlands or lowlands) and (d) altitudinal dispersal limitation caused by local adaptation. Here we simulate the evolution of clades on a mountain-like landscape where niche width and diversification rates are uniform or vary with elevation (increasing or decreasing). In our model, limitations to range expansion due to geography, niche width, and local adaptation cause variation, across elevational bands in the rate of species accumulation and altitudinal dispersal. With our simulation approach, we measure variation in strength and forms of EDG under different evolutionary scenarios, describe the expected dynamics of effective immigration across elevations, and report resulting differences in range size between highland and lowland lineages. This work generates new theoretical expectations, filling a gap in our understanding of drivers of EDGs, and expands our ability to diagnose process from patterns in empirical distributions that might be related to large-scale environmental changes. Finally, we fit dynamic likelihood models to three mountainous endemic radiations to test the theoretical predictions of our models and to empirically disentangle the contribution of diversification and dispersal to the creation of EDGs in reality.

## Methods

### Population-based model

**Figure 1.**
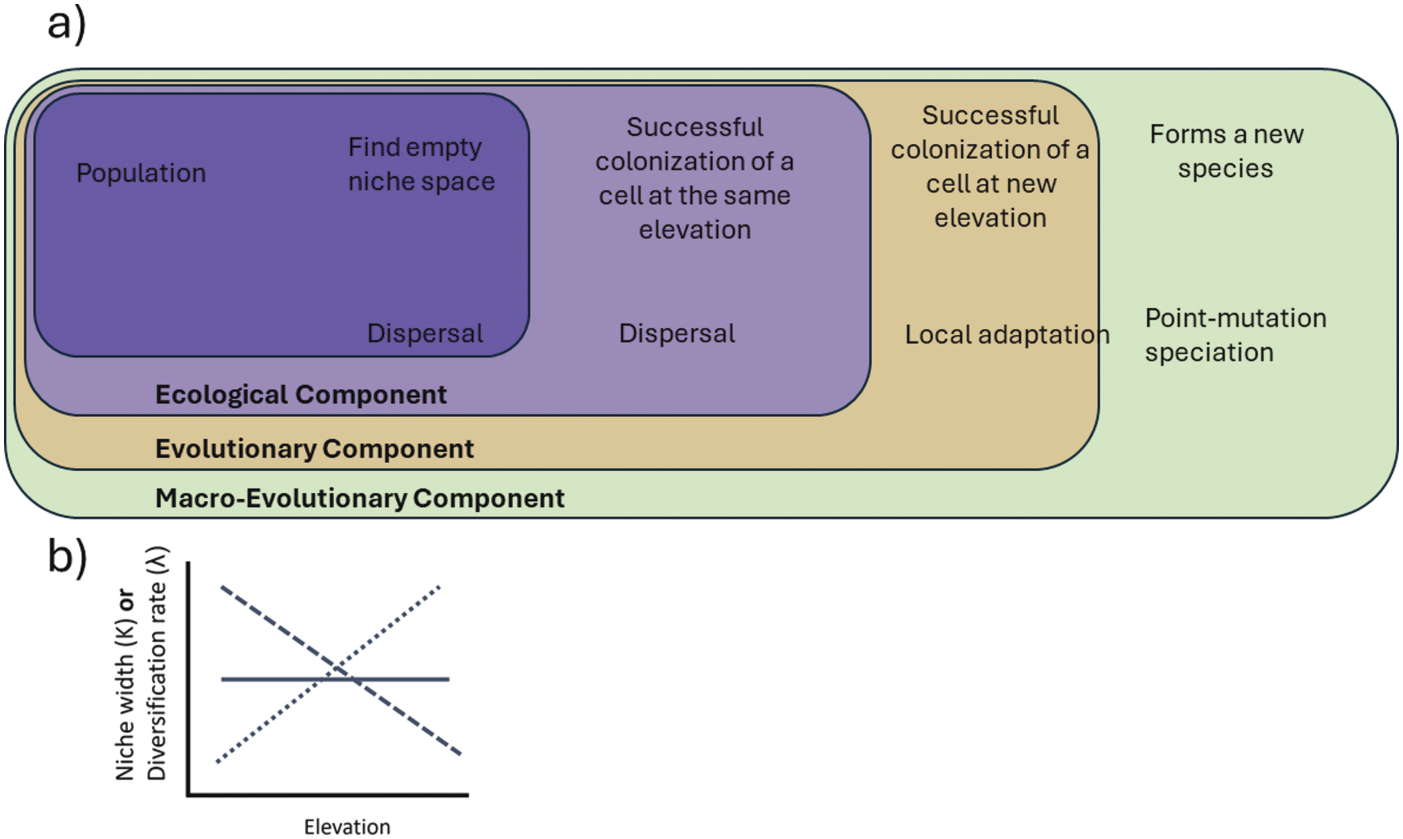
Schematic diagram of processes taking place in our model across ecological and evolutionary dimensions (A). We set different scenarios where per-capita rates of diversification as well as niche width could independently increase, decrease or be uniform with elevation (B).

We simulated the radiation of a clade across an elevational gradient using a population-based model. In our model, species’ range expansion is influenced by the interaction of mountain geometry with elevational niche availability, and local adaptation. Furthermore, mountain geometry interacts with differential rates of diversification. All these processes in turn change the rates of diversity accumulation and altitudinal dispersal along the gradient (Figure 1A). Below, we describe the abiotic and evolutionary components of the model.

#### Processes and dynamics during simulation

The populations in our model undergo four basic processes: geographic range expansion, local adaptation, geographic contraction, and diversification (Herrera-Alsina et al., 2018, 2021) taking place in a gridded domain that lies over a coned-shaped landscape (see below). A species will expand its geographic range by dispersing to new cells (at rate *γ*). The cells available for colonization are those that are adjacent to the species range and whose niche width is not saturated. A cell’s saturation is defined as the maximum number of different species that a cell can hold: species-level carrying capacity *K*. Species contract their range by going locally extinct (extirpation) from a cell at rate *μ*. When the last population of a species undergoes extirpation, the species is extinct. Diversification in our model is simplified and represents the net increase in species in the system. We modeled diversification as the creation of new species by taking one population from the parental species to then become a new species, so that all species start with range size of one cell. This process can be seen as mutation resulting in a speciation event. The rate of diversification *λ* and ecological niche width *K* are determined by the environment (a property of the elevation at which the population is located). Each population, moreover has a temperature preference, which is inherited during diversification but evolves with each colonization event. The colonizing population will have a temperature preference slightly different from its parental population: adding or subtracting a random value taken from a normal distribution of mean 0 and standard deviation of 1. We implemented local adaptation as the dependence of *γ* on the difference between the temperature preference of a population (*T*_*p*_) and the temperature at a given cell (*T*_*c*_; Bocedi et al., 2013).


$$\begin{array}{l} exp\left(-\left(\frac{{\left(T_{p}-T_{c}\right)}^{2}}{2V}\right)\right) \end{array}$$

where *V* represents the strength of stabilizing selection and local adaptation, and it determines population’s fitness and therefore how steeply population survival declines as it moves away from its best-adapted condition. Selection is strong when *V* = 5, while it weakens when *V* is large (> 1,000), effectively corresponding to absence of local adaptation. Notice that because the rates of the four basic processes are defined at population level, two species will differ in total rates (per-lineage rates) if they differ in the number of populations. As for *λ* and *K*, *T*_*c*_ is programmed to vary with elevation (see below).

#### Landscape

The landscape is a gridded domain in cone shape (similar to many mountains (Elsen & Tingley, 2015); Supplementary Figure S1) with four concentric elevational bands. In nature, the decrease in area with elevation can vary widely, from exponential to linear relationships (Elsen & Tingley, 2015). Here, we represented this decrease through a reduction in the number of cells towards the mountaintop, where highlands represent 5.8% of the total area and lowlands 58%. The intermediate bands represent 24% and 13%. Because temperature decreases with elevation, cells have a temperature value, ranging from 20° in lowlands and decreasing 5° at each elevational band. Notice that changes in how steep the temperature gradient is, would increases/decreases the simulated time needed for species to show signal of local adaptation (see below). The landscape also featured gradients in per-lineage rates of diversification by varying diversification (*λ*) according to elevation: We modeled different scenarios where *K* and *λ* increase, decrease or are uniform with elevation (Figure 1B). The difference in *λ* between the most speciose and least speciose elevational band was nine-fold and for *K*, the elevational band with the widest niche could hold four times more species locally than the band with the narrowest niche. To further disentangle the effects of dissimilar area across elevation (i.e., cone shape) from the influence of highlands being surrounded by lowlands, we run the simulations on a landscape where the mountaintop is flat and extensive (i.e., plateau where highlands represent 40% of the total area, intermediate bands represent 16%–19% whereas lowlands represent 20%; Supplementary Figure S1). This additional scenario provides insights on diversity patterns in plateau-shaped mountains (Elsen & Tingley, 2015).

#### Simulation initialization, equilibrium and variables being tracked

Simulations started with a single population randomly placed in either lowlands or highlands; the temperature preference of the population matches the temperature of that cell. Simulations run in continuous time, and the waiting times between events are randomly taken from an exponential distribution whose parameter is the total number of populations times the rates of colonization, extirpation and diversification (Gillespie algorithm, Gillespie, 1977). At the beginning of the simulation, when the landscape is empty, populations can easily disperse so that local saturation does not limit species’ range expansion. In this stage, range expansion is only constrained by local adaptation (see below) and both local and regional richness increase over time until most of cells are at *K* (Herrera-Alsina et al., 2018). At this point, local richness cannot increase, but species keep accumulating at regional scale because of turnover (non-equilibrium). We let the simulation run until we ensure that regional richness stops increasing (by visualizing the species accumulation curve over time; Supplementary Figure S4), which indicates a dynamic equilibrium. For those simulations where local adaptation is turned on, the system could also be at (non-) equilibrium in local adaptation to temperature; thus, we kept track of the mismatch between *T*_*c*_ and *T*_*p*_ over time. We found that an equilibrium in local adaptation (i.e., no further change in average *T*_*c*_ − *T*_*p*_) is attained earlier than equilibrium in regional richness, and we used the latter to define stages of equilibrium and non-equilibrium. When simulations ended, we retrieved patterns of biodiversity distribution, altitudinal dispersal across four elevational bands, and intra-elevational band variation in temperature preference recorded for the entire duration of the simulation.

#### Evolutionary scenarios

We defined three scenarios of association between *K* and elevation: niche width decreases with altitude (lowlands can pack more species at each cell than highlands), niche width increases with altitude (lowlands can pack less species at each cell than highlands), and uniform niche width in altitude. Similarly, we set three scenarios where per-lineage diversification rate varied with elevation: *λ* is higher in lowlands than highlands, *λ* is lower in lowlands than highlands, and *λ* uniform across elevational bands. With the nine combinations of *K* and *λ* we ran sets of simulations where local adaptation was turned on and where it was turned off. Simulations were run with highland or lowland origin (i.e., the location of the first population), yielding to a total of 36 different scenarios; we ran 50 replicates for each. Finally, we carried out the simulations in both landscapes (cone- and plateau-shaped). To explore whether our results remain true with a different choice of parameters, we also ran simulations where the variation in diversification rates along elevation was small (i.e., high rate was twice as high as the low rate, in contrast with our main simulation where high rate was nine times higher than the low rate). We did not explore how rates of colonization and local extinction (gamma and mu, respectively) affect the outcome of the simulations because a sister model to ours has shown that (a) variation in colonization rate does not influence regional species richness, and (b) variation in local extinction rate has no effect on how richness relates to available area (Herrera-Alsina et al, 2018, Evolution Figure 6). Simulations are coded in R and c++ (c++ is integrated into R using rcpp package) and are available at https://doi.org/10.6084/m9.figshare.24,534,760.v1.

### Empirical datasets

Diversification and dispersal events across elevation leave a signature on phylogenetic reconstructions that can be retrieved when using adequate models (van Els et al., 2021). When elevational bands are associated with differential diversification rates, the branch lengths of a phylogenetic tree will show systematic variation in diversification rates (i.e., the branching pattern) as lineages switch from one elevation to another. Importantly, the change in elevation undertaken by species also modifies the richness along the elevational gradient. We fit likelihood models to real-world radiations (see below) where diversification rates across elevations are simultaneously modeled with changes in elevation. In these models, termed state-dependent diversification models (Maddison et al., 2007), the probability of a species being present at a given elevation depends on (a) the diversification rate for that elevation, and (b) the rate of switching to and out this elevational band. We looked into the evolution of frailejon bushes (Pouchon et al., 2021), Fijian bees (Dorey et al., 2020) and earless frogs (Von May et al., 2018), which are endemic mountainous monophyletic clades (similar to our simulated ones), and whose diversity peaks in intermediate or high-elevation. We used phylogenetic trees and elevation data provided at the original publications. We classified species’ elevation into lowland, midland and highland species by defining three elevational bands of equal width bounded by the lowest and highest elevations recorded for species. This categorization is necessary because statistical-robust likelihood methods cannot handle continuous variables (Beaulieu & O’Meara, 2016; Herrera-Alsina et al., 2019; Rabosky & Goldberg, 2015). We fitted likelihood-based diversification models that differ in their assumptions, compared their likelihoods using AIC weights, and recovered the parameter estimates (diversification and transition rates, see below) of the best performing model. The transition of a lineage from one elevational band to another was modeled in five ways: (a) shifting to an adjacent elevational band, uphill and downhill movements happening at the same rate, (b) shifting to an adjacent elevational band, uphill and downhill movements happening at different rates, (c) shifting to any elevational band, uphill and downhill movements happening at the same rate, (d) shifting to any elevational band, uphill and downhill movements happening at different rates, and (e) each movement into and out of any elevation has its own rate. We modeled changes in diversification rates that are either dependent or independent of elevational shifts. In elevational-dependent models, we did not assume any elevational band to have increased diversification rate, instead, we allowed the model to estimate which band has the highest rate. We started the likelihood maximization of the 15 models in three different points of the parameter space to avoid finding only local optima. We used the R package SecSSE (Herrera-Alsina et al., 2019) for this analysis.

## Results

### Simulation models

**Figure 2.**
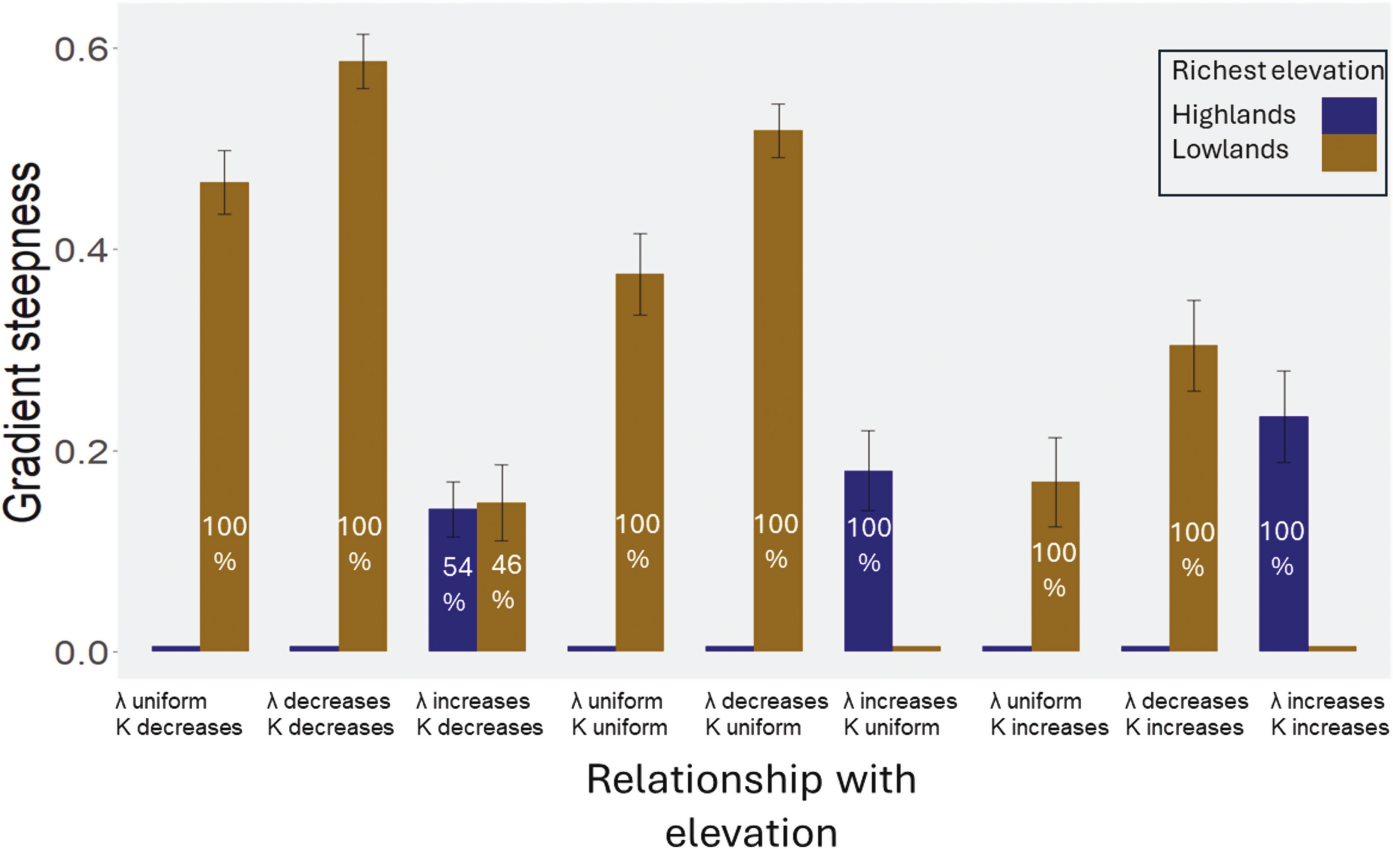
EDG changes in strength and directionality across different simulated conditions. We simulated nine scenarios (50 replicates each) where per-capita diversification rate (*λ*) and niche width (*K*) decreased, increased, or were kept uniform with elevation. In eight scenarios, 100% of the replicates (in white font) showed that richness peaks either at lowlands (brown bar) or highlands (blue bar). However, when *λ* increases and *K* decreases (third scenario from the left) 54% of simulations peaked in highlands and the rest in lowlands. *Y*-axis shows the average gradient strength (the relative difference in species richness from the richest band to the next) across replicates along with error bars (whiskers).

In the absence of differences in niche width (*K*) and diversification rates across elevation (i.e., geometry is the sole factor), we found that species richness decreases with altitude, with lowlands being the most species-rich (Figure 2). This gradient in species richness is increased when niche width is no longer uniform (geometry + *K*) but higher in lowlands than highlands and decreased when niche width varies in the opposite direction (Figure 2). However, the influence of *K* is never strong enough to entirely counteract the impact of geometry on species richness. Local adaptation yielded, in general, to similar diversity patterns as models with no local adaptation (see section below). Interestingly, when simultaneously considering geometry and variation in diversification rates (i.e., under uniform *K*), the elevational band with the highest diversification rates is the one showing the highest species richness is. Species richness peaks at mid- or high elevations when highlands boast high diversification rates compared to lowlands. This suggests that a positive relationship between diversification rate and altitude effectively counteracts the effects of geometry in species accumulation (Figure 2). However, this does not hold in simulations where the difference between the lowest and the highest diversification rates is small (Supplementary Figure S2). To investigate the interaction between geometry, *K* and diversification rates we had two scenarios: lowlands-have-it-all where both diversification rate and niche width are higher in lowlands than highlands and highlands-have-it-all with the opposite configuration. Our simulations show that in lowlands-have-it-all, lowlands are the richest with the steepest decrease in diversity with altitude. In highlands-have-it-all, the midlands are the richest elevation, and the EDG is of moderate intensity. Interestingly, the steepness of the gradient resulting in the highlands-have-it-all scenario is no different from the gradient resulting when highlands have high diversification rates, but *K* is uniform. The insights of these results are threefold: (a) diversification rate variation across altitude exerts higher influence on elevational patterns of diversity than niche width variation does, (b) richness can increase with elevation, but the EDG will never be steep, and (c) lowland diversity is impacted by the interaction between *K* and diversification rate whereas highland diversity is not.

**Figure 3.**
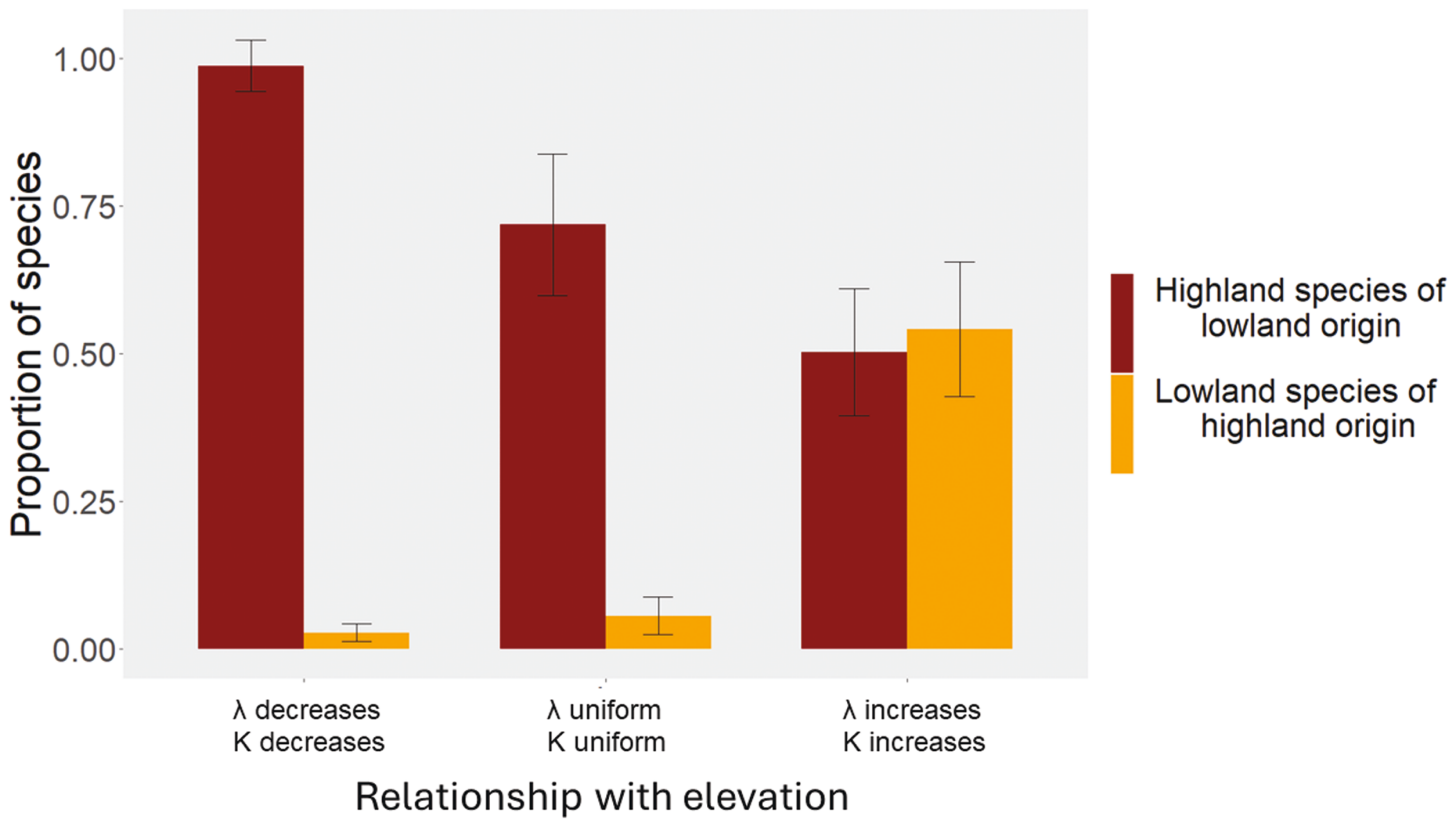
The proportion of species at a given elevation that originated at a different elevational band. Bar height shows the average proportion across replicates along with error bars (whiskers). We show three scenarios (with 50 replicates each) which differed in where per-capita diversification rate (*λ*) and niche width (*K*) are the highest: lowlands (left panel), highlands (right panel) or uniform (middle panel). A similar plot to this but featuring local adaptation is available in Supplementary Material.

When looking at the net dispersal across elevational bands, our results show that when geometry is the sole factor affecting the EDG (i.e., uniform niche width and diversification rates along elevation), the contribution of lowlands to highland diversity is high: a large proportion of lineages found in highlands are in fact, of lower elevational origin (Figure 3). Lowland diversity, on the other hand, is mostly formed by lineages that originated at this elevation with a small contribution from lineages originated at higher elevation. Lineages tend to move across elevations more often before the system equilibrates: lowlands and highlands receive more dispersers when the clade is young (Supplementary Figure S3). The only scenario where the number of downhill dispersers is similar to the number of species moving to higher elevation is when both rate of diversification and niche width are higher in highlands than lowlands (i.e., highlands-have-it-all) (Figure 3).

In our plateau-shaped landscape (where the large-area highlands are surrounded by narrow bands of lower elevation; Supplementary Figure S1), the highest elevation is double in surface than lowlands and yet, our model predicts that diversity will peak in lowlands when *K* and diversification rates are uniform. This is because the movement of species across different locations *within* an elevation band is slower in lowlands than in a plateau, as while lowlands are structured in a “ring,” cells in a plateau are better connected to each other. This difference in intra-elevation dispersal in turn causes species turnover and richness to be higher in lowlands. Therefore, our results suggest that while available area is a key factor driving the dynamics of species accumulation over time, the space configuration is also important.

We calculated the proportional occupied area for every species at each elevational band by measuring the species’ range size and dividing it by the total area available at a given band. High-elevation species tend to occupy a large proportion of the available area (40% in average) in comparison to lowland species (8% in average), and this pattern is more pronounced when highlands have low rates of diversification. However, when niche width at highest elevation is low, range sizes will be small due to limited opportunities for range expansion (i.e., local saturation is reached early). Unlike highlands, species inhabiting lowlands have range sizes that are proportionally small (when compared with the large area available at this elevation), especially when rates of diversification are high.

#### Influence of local adaptation

Simulations that featured local adaptation show that this process does not influence the strength of EDG nor its directionality, but it does impact different aspects of biodiversity. For instance, when considering the mountain system as a whole (i.e., all elevational bands), simulations with local adaptation resulted in higher regional species richness than simulations with no local adaptation (Supplementary Figure S4). Furthermore, local adaptation reduces the net flow of species across elevation, and we found the interesting emergent tendency that dispersal of highland lineages is more limited by local adaptation than lowland lineages (Supplementary Figure S5). In models with local adaptation, we found that in all scenarios, populations at a given elevation have small variation in temperature preference and this pattern is consistent at any elevation (Supplementary Figure S6). However, the only exception is when niche width increases with altitude (irrespective of how diversification rates are associated with altitude), temperature-adapted lowland populations will be more variable in temperature preference than their highland counterpart.

### Empirical endemic radiations

**Figure 4.**
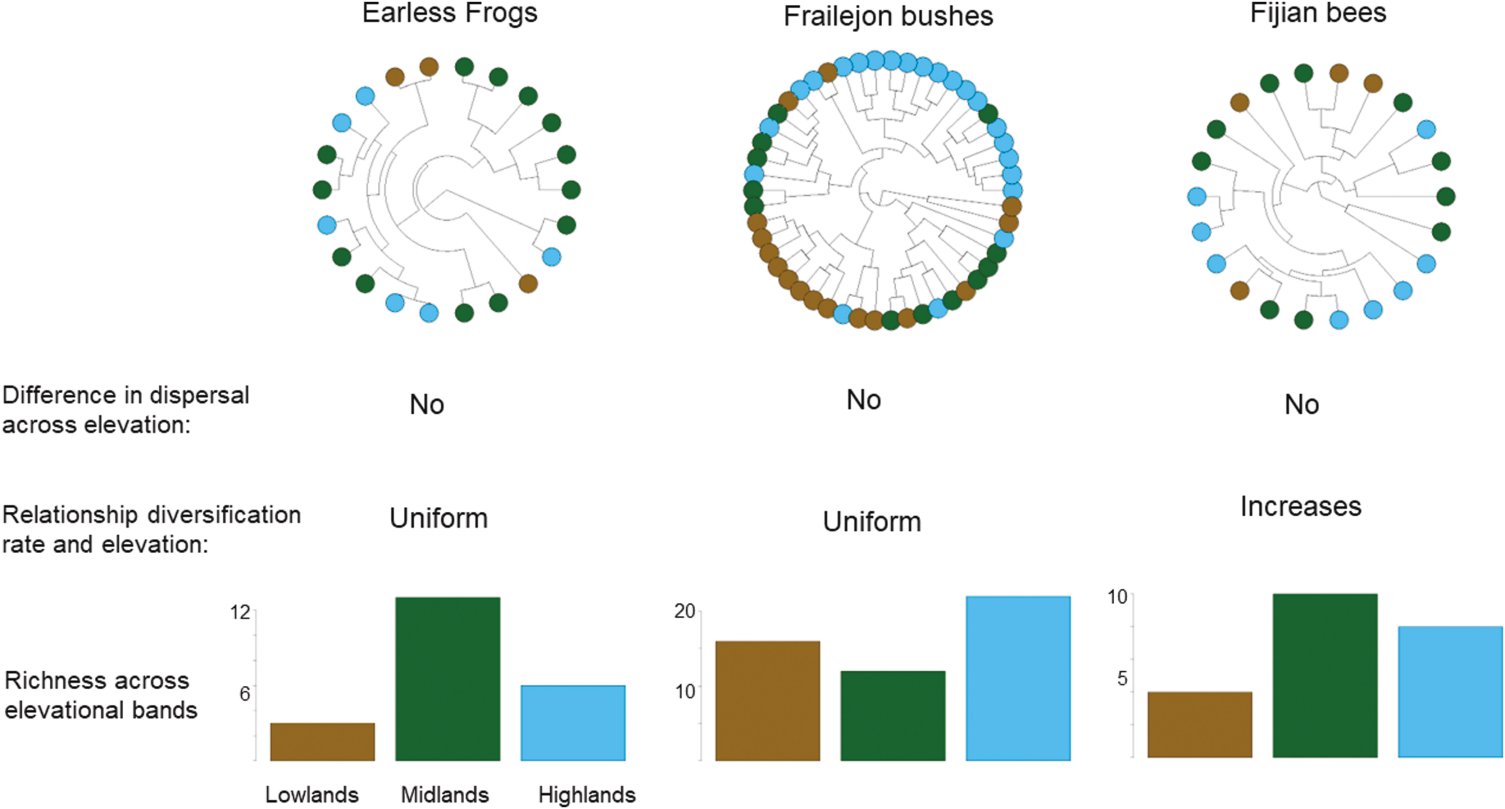
Three mountain endemic radiations where species richness peaks at mid-to high elevations. We fit maximum likelihood models that differ in the how diversification and dispersal vary across three elevational bands. This figure summarizes the results from the best supported model for each clade (see Supplementary Table S1).

In earless frogs, frailejon bushes and Fijian bees, we found high statistical support for models where downhill and uphill movement take place at the same rate (pooled AIC weights = 66%, 49% and 73% respectively; Figure 4). Moreover, models assuming that lineages only disperse to adjacent bands were better supported than models without this assumption in earless frogs and Fijian bees (Supplementary Table S1). In contrast, we found that frailejon bushes tend to move across elevations in a less restricted manner; for instance, a lowland species can shift to highlands without passing to middle elevation first. Interestingly, Fijian bees show strong support for elevation-dependent diversification (pooled AIC weights = 47%); in other words, species systematically increase their diversification rates while inhabiting the highlands and decrease their rates when moving to lower elevation (Figure 4). In contrast, models with elevation-independent diversification in earless frogs and frailejon bushes performed best: lineages diversify at the same rate at any elevational band (pooled AIC weights = 84% and 89% respectively; Supplementary Table S1). In particular, models with homogeneous diversification rates across lineages were highly supported for these two clades.

## Discussion

Our simulation predicts, just as empirical data suggests, that there is a wide range of possible outcomes for diversity patterns, which ultimately depend on mountain geometry, evolutionary (altitudinal variation in diversification rates) and ecological (altitudinal variation in niche width) factors, and clade age. Patterns in nature are complex and to disentangle the underlying mechanisms, one should contrast them with simpler, adequate theoretical expectations such as the ones we have provided.

The arrival of species *into* the system (we simulate an endemic clade in a mountain system where no immigration from elsewhere takes place) is not included in the model, but our framework allows us to describe how it might influence the distribution of species. Whether the elevational gradient changes or not in presence of dispersers from outside will depend on what stage of the clade’s evolution immigration takes place. The arrival of outside dispersers occurs early in the clade’s history when local saturation is not reached, this guarantees successful colonization at wherever elevation niche width is least occupied. If niche width is uniform, lowlands could have an increased chance of receiving migrants due to their large available area. This means that the connectivity of a mountain to an adjacent source of species can change the distribution of richness across elevation. For instance, rodent diversity in Mt. Taibai distributes in a hump-shaped fashion in the northern slope of the mountain, which is isolated by physical barriers and potentially receives few immigrants (Shuai et al., 2017). In contrast, the southern slope of the mountain is better connected to the rest of the region and shows monotonic decrease of diversity with elevation. According to our results, this distribution of species in the northern slope is consistent with scenarios where diversification rates are high at highlands. This suggests (a) the erosion of the natural hump-shaped distribution of rodents in the southern slope via the addition of outside dispersers into the lowlands (Fu et al., 2006), and (b) evolutionary dynamics of Mt. Taibai were not in equilibrium by the time species immigration took place.

For the three empirical endemic radiations where we fit likelihood models, we found that the underlying mechanisms behind diversity peaking at intermediate or high-elevation can be different. In the case of Fijian bees, high rates of diversification at the top of the mountains guarantee that diversity does not mainly accumulate at low elevation, even if altitudinal dispersal is symmetric. The contribution of diversification counteracts mountain geometry to impact the distribution of Fijian bees, as diversification by our simulation model where we found that lowlands would be the richest unless highlands have high rates of diversification. For frailejon bushes and earless frogs, with species richness not being the highest in lowlands, we find no evidence that lowlands have low rates of diversification or that uphill dispersal events outnumber the opposite movement. This is an expected outcome from our simulation when the system has not reached a dynamic equilibrium, which is likely to be the case in these young clades. This idea is further supported by our finding that inter-lineage variation in diversification rates for both clades is rather negligible, meaning that lineages have similar rates of diversification, which matches the early stages of our simulation approach, as local saturation has not been reached in a young clade, species have similar range siz,es and diversification rates. Areas of recent mountain uplift that have driven recent radiations of clades resulting in diversity peaks above the lowlands likely experience such processes (C. Hughes & Eastwood, 2006; Pérez-Escobar et al., 2017).

In our model, reproductive isolation is not considered, but our simulations show two patterns, which are in line with previous studies where narrower thermal tolerances in the tropics than in temperate areas decrease geneflow, which promotes reproductive isolation and ultimately species divergence (Gill et al., 2016; Polato et al., 2018; Sheldon et al., 2018). In our model (a) total mountain richness in simulations featuring local adaptation was higher than simulations without this process, and (b) that highlands and lowlands are less likely to exchange lineages in presence of local adaptation. With local adaptation, species in our model take longer to be able to colonize adjacent elevational bands, so that lowland species tend to stay longer in lowlands where they can attain larger range sizes and increase their total probabilities for diversification. Thus, we found a similar macroevolutionary trend but with a different mechanistic cause (Ghalambor, 2006). Furthermore, this may be akin to patterns identified in the lowland neotropics where species with large range sizes can show considerable genetic divergence and wide-ranging speciose clades have diversified rapidly and recently (Melo et al., 2018; Richardson et al., 2001).

Our simulations show that local adaptation keeps species restricted to a given temperature resulting in large range sizes at given elevation with a subsequent increase in opportunities for diversification. The limited intra-annual variation in temperature (i.e., low seasonality) in tropical mountain systems causes that summer temperatures in highlands never occur in wintertime in the lowlands. This mechanism strengthens the effects of local adaptation by limiting inter-elevation dispersal. However, the increase in seasonality in the tropics is one of the effects of climate (Feng et al., 2013). This means that species that handle strong temperature fluctuations within a year are no longer restricted to only one elevational band. Consequently, species in the tropics are less likely to experience the range-limiting effects of local adaptation, resulting in a low accumulation of diversity.

With the ongoing climate change crisis, one of the main concerns is species’ response to it. Recent, global temperature rise has pushed species towards higher elevations to track their temperature requirements (Moritz et al., 2008; Wilson et al., 2005), with many plant and animal species altering their elevational distribution (Chen et al., 2011; Lenoir et al., 2020; Parmesan et al., 2003). While most of them have moved to higher elevations, an important percentage has moved in the opposite direction, lowering their altitudinal range (Lenoir et al., 2020). Our model shows that upslope migration should exceed downslope movements even in the absence of environmental gradients or change. This suggests that analyses of contemporary range shifts, which show predominant upslope movement of species under climate change (Lenoir et al., 2020), should consider applying more sophisticated null models that account for these expected equilibrium dispersal asymmetries across elevations (Jezkova & Wiens, 2016).

A concerning interpretation of our finding that lineages naturally tend to disperse to higher elevation, is that healthy highland biodiversity depends on lowland conservation. Lowlands and mountain foothills are especially targeted by human activities, which threatens evolutionary processes across the entire elevation gradient in mountains. Our results reinforce the paramount role of ecotones and transitional vegetation across elevational bands to facilitate the movement of lineages (Erdős et al., 2018; Wehling & Diekmann, 2009). Conservation efforts should maximize the well-being of those transitional ecosystems, particularly in young radiations, when according to our results, dispersal is at its highest rate.

## Supplementary material

Supplementary material is available online at *Evolution Letters*.

qrae048_suppl_Supplementary_Figures_Tables

qrae048_suppl_Supplementary_Appendix

qrae048_suppl_Supplementary_Data

## Data Availability

Data and code for simulation and analysis is available at: https://doi.org/10.6084/m9.figshare.24534760.v1
