# Supplementary material for: Disentangling evolutionary, geometric and ecological components of the elevational gradient of diversity
Source: Evol Lett. 2024 Sep 22;9(1):51–64. doi: 10.1093/evlett/qrae048 (PMC11790213; doi:10.1093/evlett/qrae048)
Supplement: qrae048_suppl_Supplementary_Figures_Tables [file qrae048_suppl_supplementary_figures_tables.docx]

Supplementary Material


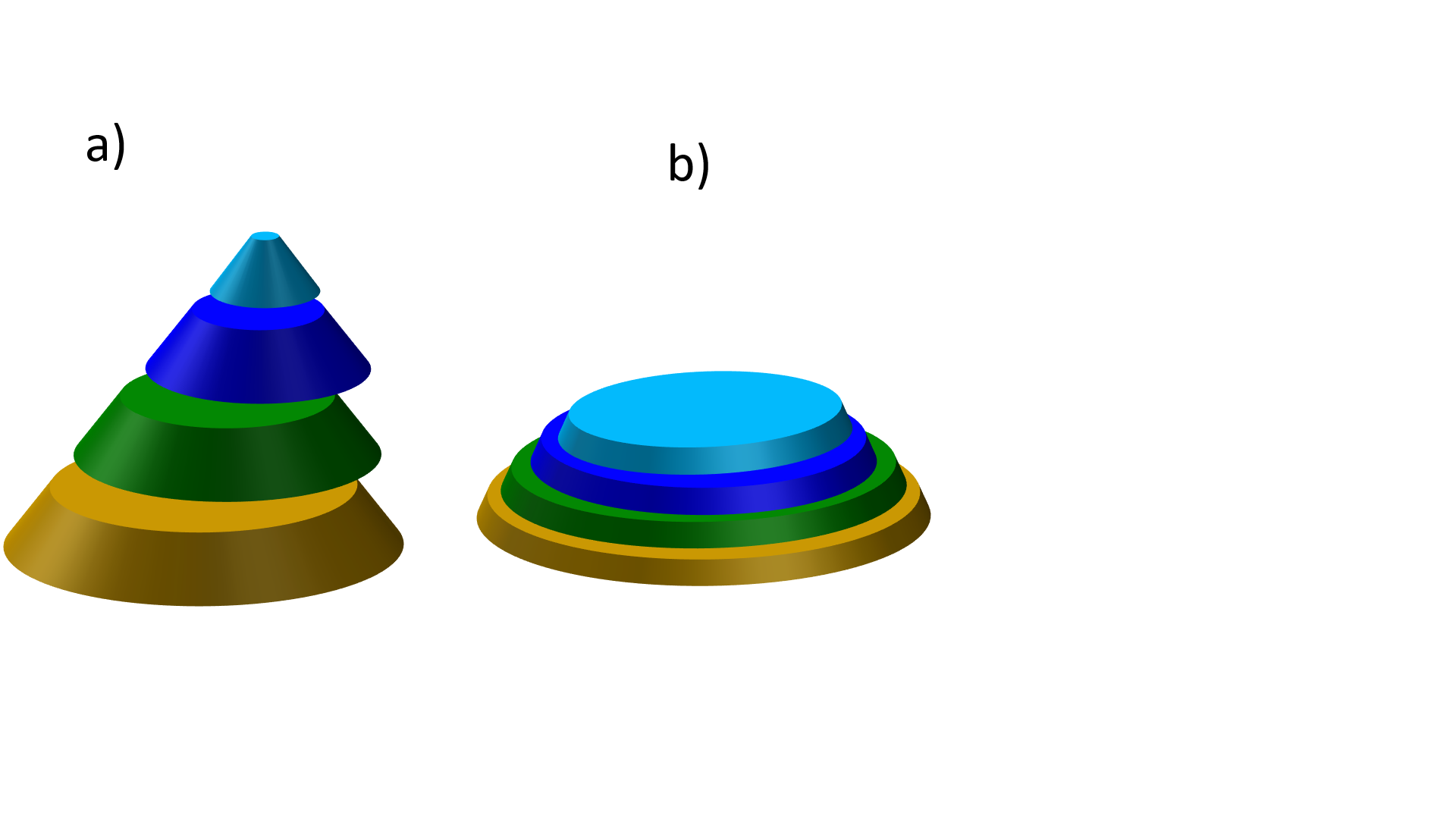


Figure S1. Two different landscapes used in our simulation: a) cone-shaped and b) plateau-shaped.


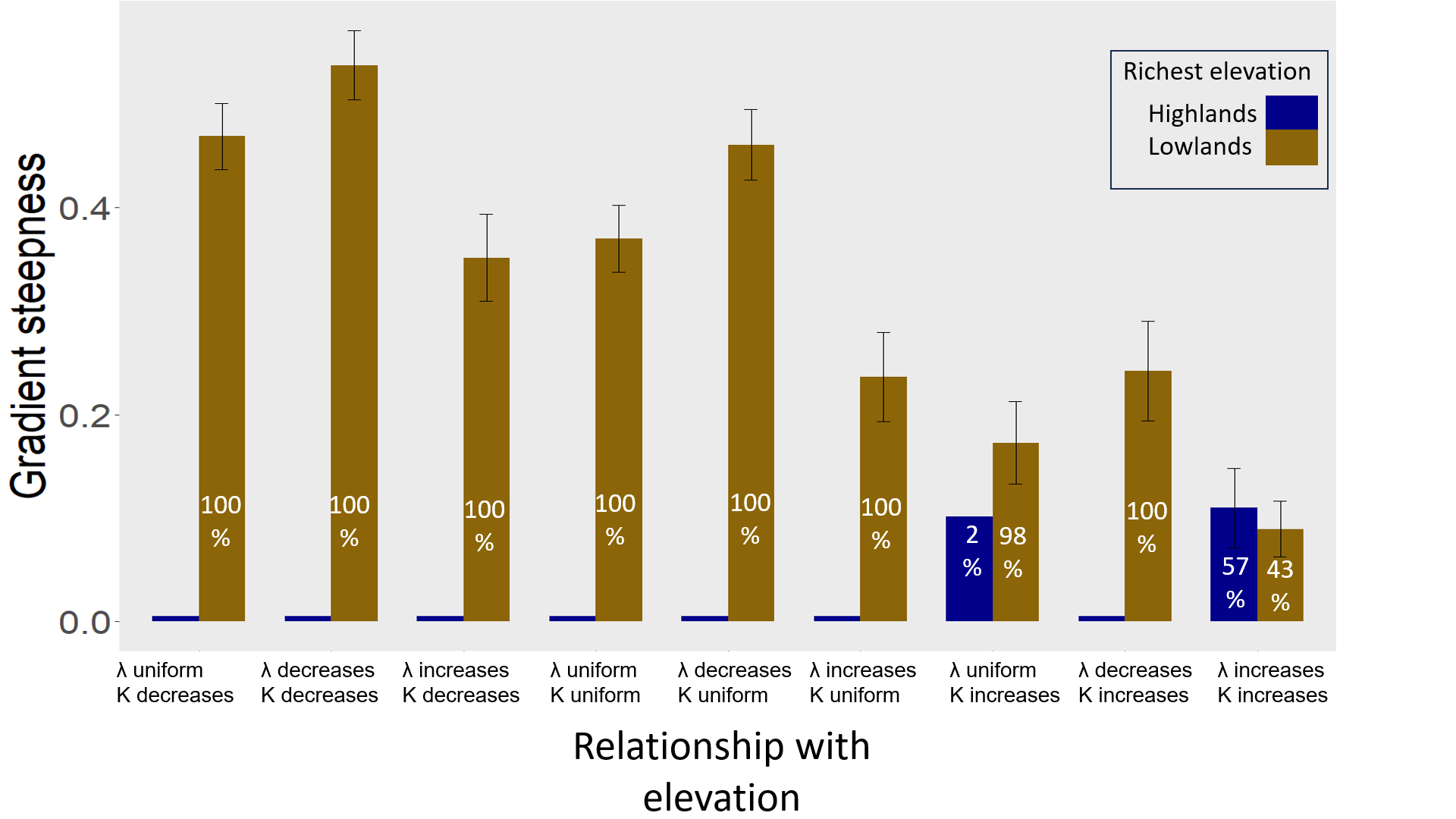


Figure S2. Elevational diversity gradient changes in strength and directionality across different simulated conditions. We simulated nine scenarios (50 replicates each) where per-capita diversification rate (λ) and niche width (K) decreased, increased, or were kept uniform with elevation. This figure shows a two-fold difference between the elevational band with the highest and the elevational band with lowest speciation rate (unlike the nine-fold difference shown in Figure 2 main text). Different from the main text where the difference between the lowest and the highest speciation rate is nine-fold, this figure shows that in eight scenarios, 100% of the replicates (in white font) showed that richness peaks either at lowlands (brown bar) or highlands (blue bar). However, when λ increases and K decreases (third scenario from the left) 54% of simulations peaked in highlands and the rest in lowlands. Y-axis shows the gradient average strength (the relative difference in species richness from the richest band to the next) across replicates along with error bars (whiskers.


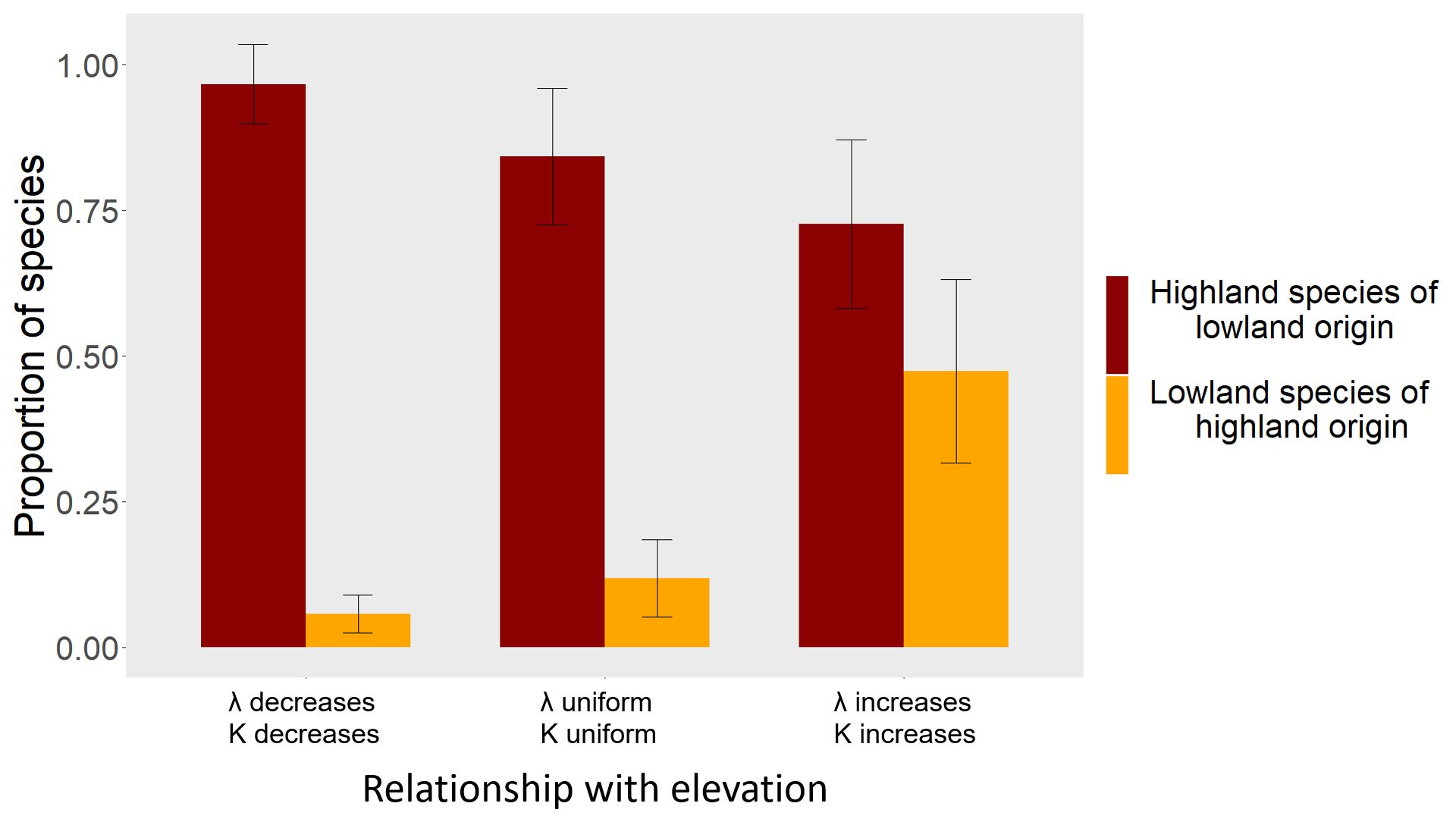


Figure S3. The proportion of species at a given elevation that originated at a different elevational band in a young clade. Bar height shows the average proportion across replicates along with error bars (whiskers). We show three scenarios (with 50 replicates each) which differed in where per-capita diversification rate (λ) and niche width (K) are the highest: lowlands (left panel), highlands (right panel) or uniform (middle panel).


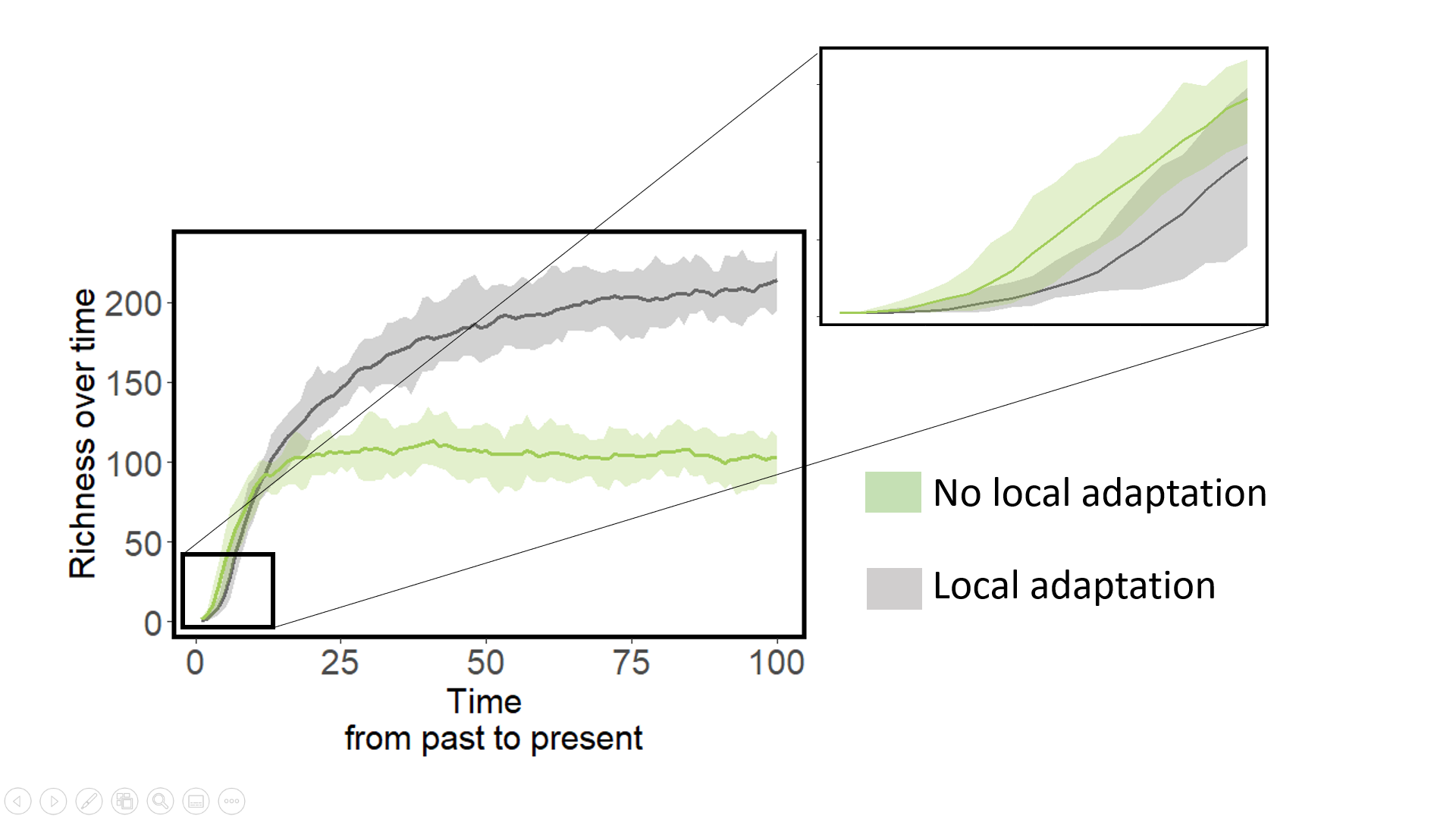


Figure S4. The accumulation of total diversity in a mountain depends on whether local adaptation is featured in the simulation or not. We modelled the evolution of a clade since its origin and over 100 units of simulated time (x-axis) and show the average number of extant species at each point in time across 50 simulations (y-axis, solid lines). 95% intervals are shown as shaded areas.


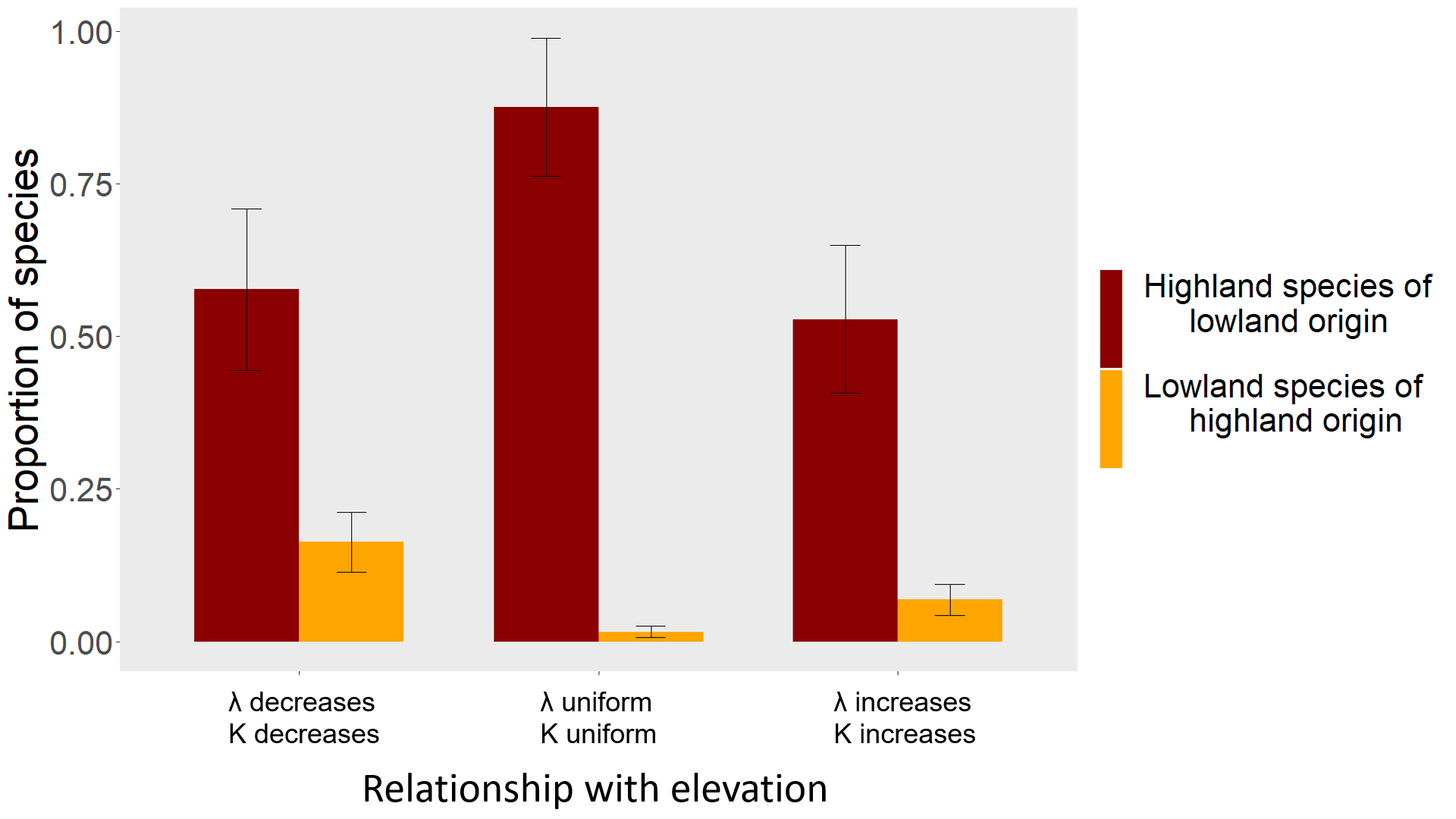


Figure S5. Dispersal of lineages across elevational bands when local adaptation is switched on. Bars indicate the proportion of species at a given elevation that are originated at a different elevational band. We show three scenarios (with 50 replicates each) which differed in where per-capita diversification rate (λ) and niche width (K) are the highest: lowlands (left panel), highlands (right panel) or uniform (middle panel).


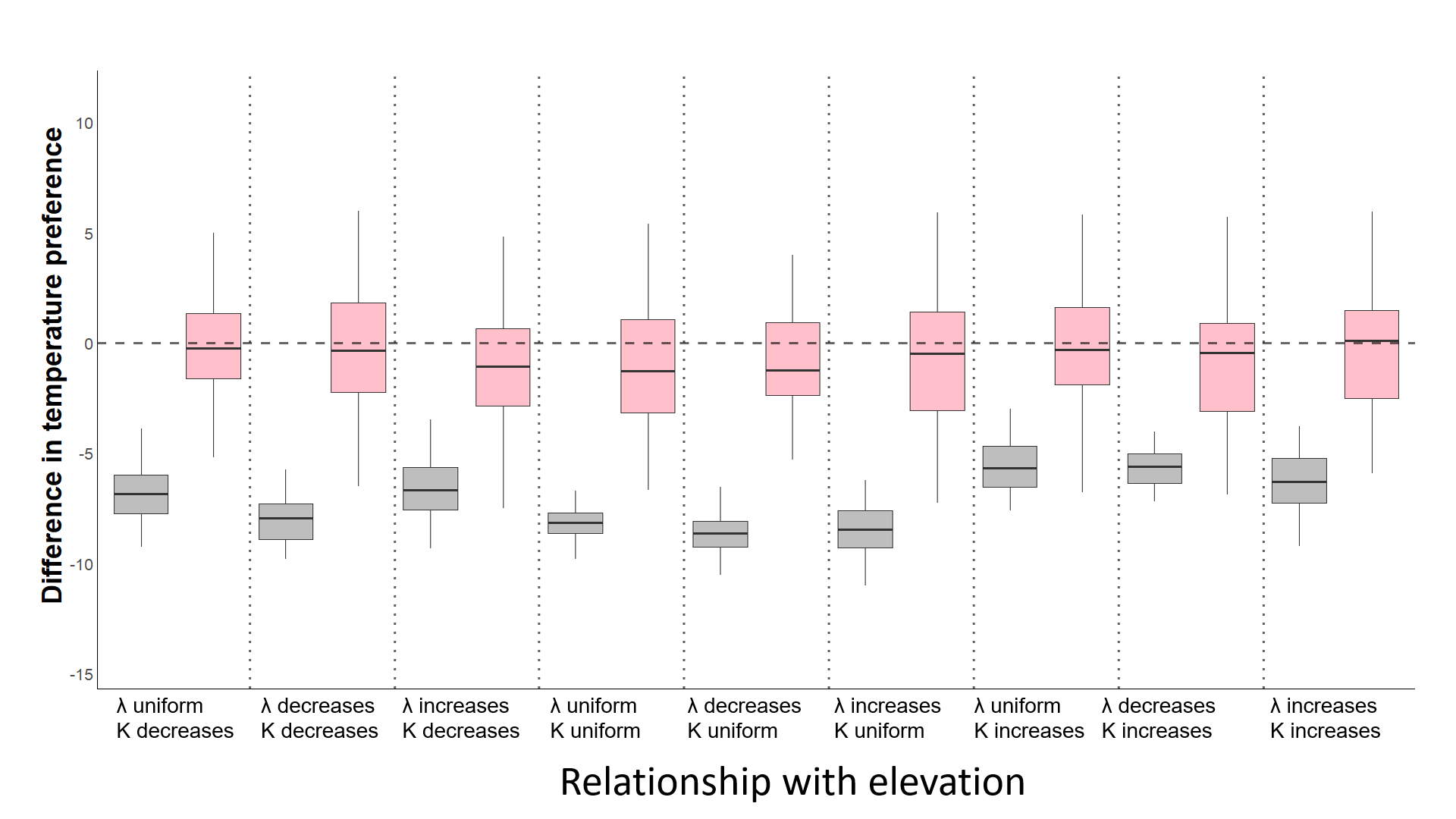


Figure S6. Variation in evolved temperature preference across all highland and lowland populations. Pink boxes show simulations where local adaptation is absent and gray boxes show simulations featuring local adaptation. In x axis we show nine scenarios (50 replicates each) where per-capita diversification rate (λ) and niche width (K) decreased, increased, or were kept uniform with elevation.

Table S1. Summary of assumptions and statistical support values for 15 models fitted to three endemic radiations.

| Relationship diversification and elevation | Dispersal across elevation | (log) Likelihood | Number of free parameters | AICweights |
| --- | --- | --- | --- | --- |
| **Earless frogs** |  |  |  |  |
| Elevation-independent diversification. Homogeneous rates across species | Adjacent band dispersal- single rate | -72.949 | 3 | 0.28 |
| Elevation-independent diversification. Homogeneous rates across species | Any band dispersal- single rate | -73.195 | 3 | 0.22 |
| Elevation-independent diversification. Homogeneous rates across species | Adjacent band dispersal- up/down rates | -72.900 | 4 | 0.11 |
| Elevation-independent diversification. Homogeneous rates across species | Any band dispersal- up/down rates | -73.076 | 4 | 0.09 |
| Elevation-dependent diversification | Adjacent band dispersal- single rate | -72.497 | 5 | 0.06 |
| Elevation-dependent diversification | Any band dispersal- single rate | -72.765 | 5 | 0.05 |
| Elevation-independent diversification. Homogeneous rates across species | Any band dispersal- six rates | -69.769 | 8 | 0.04 |
| Elevation-independent diversification. Heterogeneous rates across species | Adjacent band dispersal- single rate | -72.949 | 5 | 0.04 |
| Elevation-independent diversification. Heterogeneous rates across species | Any band dispersal- single rate | -73.195 | 5 | 0.03 |
| Elevation-dependent diversification | Adjacent band dispersal- up/down rates | -72.271 | 6 | 0.03 |
| Elevation-dependent diversification | Any band dispersal- up/down rates | -72.722 | 6 | 0.02 |
| Elevation-dependent diversification | Any band dispersal- six rates | -68.795 | 10 | 0.02 |
| Elevation-independent diversification. Heterogeneous rates across species | Adjacent band dispersal- up/down rates | -72.900 | 6 | 0.01 |
| Elevation-independent diversification. Heterogeneous rates across species | Any band dispersal- up/down rates | -73.076 | 6 | 0.01 |
| Elevation-independent diversification. Heterogeneous rates across species | Any band dispersal- six rates | -69.761 | 10 | 0.01 |
| **Frailejon bushes** |  |  |  |  |
| Elevation-independent diversification. Homogeneous rates across species | Any band dispersal- single rate | -59.718 | 3 | 0.25 |
| Elevation-independent diversification. Homogeneous rates across species | Any band dispersal- up/down rates | -59.285 | 4 | 0.14 |
| Elevation-independent diversification. Heterogeneous rates across species | Any band dispersal- single rate | -58.496 | 5 | 0.11 |
| Elevation-independent diversification. Heterogeneous rates across species | Any band dispersal- up/down rates | -57.629 | 6 | 0.10 |
| Elevation-independent diversification. Heterogeneous rates across species | Any band dispersal- six rates | -53.630 | 10 | 0.10 |
| Elevation-independent diversification. Homogeneous rates across species | Any band dispersal- six rates | -55.775 | 8 | 0.09 |
| Elevation-independent diversification. Homogeneous rates across species | Adjacent band dispersal- single rate | -61.184 | 3 | 0.06 |
| Elevation-dependent diversification | Any band dispersal- single rate | -59.548 | 5 | 0.04 |
| Elevation-dependent diversification | Any band dispersal- up/down rates | -58.805 | 6 | 0.03 |
| Elevation-independent diversification. Heterogeneous rates across species | Adjacent band dispersal- single rate | -60.100 | 5 | 0.02 |
| Elevation-independent diversification. Homogeneous rates across species | Adjacent band dispersal- up/down rates | -61.107 | 4 | 0.02 |
| Elevation-dependent diversification | Any band dispersal- six rates | -55.204 | 10 | 0.02 |
| Elevation-independent diversification. Heterogeneous rates across species | Adjacent band dispersal- up/down rates | -59.604 | 6 | 0.01 |
| Elevation-dependent diversification | Adjacent band dispersal- single rate | -60.839 | 5 | 0.01 |
| Elevation-dependent diversification | Adjacent band dispersal- up/down rates | -60.751 | 6 | 0.00 |
| **Fijian bees** |  |  |  |  |
| Elevation-dependent diversification | Adjacent band dispersal- single rate | -11.157 | 5 | 0.23 |
| Elevation-dependent diversification | Any band dispersal- single rate | -11.282 | 5 | 0.20 |
| Elevation-independent diversification. Heterogeneous rates across species | Adjacent band dispersal- single rate | -11.880 | 5 | 0.11 |
| Elevation-independent diversification. Homogeneous rates across species | Adjacent band dispersal- single rate | -14.128 | 3 | 0.09 |
| Elevation-dependent diversification | Any band dispersal- up/down rates | -11.275 | 6 | 0.07 |
| Elevation-independent diversification. Heterogeneous rates across species | Adjacent band dispersal- up/down rates | -11.481 | 6 | 0.06 |
| Elevation-independent diversification. Heterogeneous rates across species | Any band dispersal- single rate | -12.572 | 5 | 0.06 |
| Elevation-independent diversification. Homogeneous rates across species | Any band dispersal- single rate | -14.703 | 3 | 0.05 |
| Elevation-independent diversification. Homogeneous rates across species | Adjacent band dispersal- up/down rates | -14.007 | 4 | 0.04 |
| Elevation-independent diversification. Heterogeneous rates across species | Any band dispersal- up/down rates | -12.063 | 6 | 0.03 |
| Elevation-independent diversification. Homogeneous rates across species | Any band dispersal- up/down rates | -14.211 | 4 | 0.03 |
| Elevation-dependent diversification | Any band dispersal- six rates | -9.010 | 10 | 0.01 |
| Elevation-dependent diversification | Adjacent band dispersal- up/down rates | -13.030 | 6 | 0.01 |
| Elevation-independent diversification. Heterogeneous rates across species | Any band dispersal- six rates | -10.196 | 10 | 0.00 |
| Elevation-independent diversification. Homogeneous rates across species | Any band dispersal- six rates | -13.233 | 8 | 0.00 |
