# Supplementary material for: Disentangling evolutionary, geometric and ecological components of the elevational gradient of diversity
Source: Evol Lett. 2024 Sep 22;9(1):51–64. doi: 10.1093/evlett/qrae048 (PMC11790213; doi:10.1093/evlett/qrae048)
Supplement: qrae048_suppl_Supplementary_Appendix [file qrae048_suppl_supplementary_appendix.docx]

Appendix 2. Reference list for studies in Table 1

Abebe, A.F., Cai, T., Wale, M., Song, G., Fjeldså, J., Lei, F., 2019. Factors determining species richness patterns of breeding birds along an elevational gradient in the Horn of Africa region. Ecology and Evolution 9, 9609–9623. https://doi.org/10.1002/ece3.5491

Acharya, B.K., Sanders, N.J., Vijayan, L., Chettri, B., 2011. Elevational gradients in bird diversity in the Eastern Himalaya: an evaluation of distribution patterns and their underlying mechanisms. PLoS One 6, e29097. https://doi.org/10.1371/journal.pone.0029097

Acharya, B.K., Vijayan, L., 2015. Butterfly diversity along the elevation gradient of Eastern Himalaya, India. Ecological Research 30, 909–919. https://doi.org/10.1007/s11284-015-1292-0

Betz, O., Srisuka, W., Puthz, V., 2020. Elevational gradients of species richness, community structure, and niche occupation of tropical rove beetles (Coleoptera: Staphylinidae: Steninae) across mountain slopes in Northern Thailand. Evol Ecol 34, 193–216. https://doi.org/10.1007/s10682-020-10036-2

Bharti, H., Sharma, Y.P., Bharti, M., Pfeiffer, M., 2013. Ant species richness, endemicity and functional groups, along an elevational gradient in the Himalayas. Asian Myrmecology 5, 79–101.

Bhatt, J.P., Manish, K., Pandit, M.K., 2012. Elevational Gradients in Fish Diversity in the Himalaya: Water Discharge Is the Key Driver of Distribution Patterns. PLOS ONE 7, e46237. https://doi.org/10.1371/journal.pone.0046237

Brehm, G., Süssenbach, D., Fiedler, K., 2003. Unique elevational diversity patterns of geometrid moths in an Andean montane rainforest. Ecography 26, 456–466. https://doi.org/10.1034/j.1600-0587.2003.03498.x

Chettri, B., Acharya, B.K., 2020. Distribution of amphibians along an elevation gradient in the Eastern Himalaya, india. Basic and Applied Ecology 47, 57–70. https://doi.org/10.1016/j.baae.2020.07.001

Cirimwami, L., Doumenge, C., Kahindo, J.-M., Amani, C., 2019. The effect of elevation on species richness in tropical forests depends on the considered lifeform: results from an East African mountain forest. Trop Ecol 60, 473–484. https://doi.org/10.1007/s42965-019-00050-z

Corcos, D., Cerretti, P., Mei, M., Vigna Taglianti, A., Paniccia, D., Santoiemma, G., De Biase, A., Marini, L., 2018. Predator and parasitoid insects along elevational gradients: role of temperature and habitat diversity. Oecologia 188, 193–202. https://doi.org/10.1007/s00442-018-4169-4

Fu, C., Hua, X., Li, J., Chang, Z., Pu, Z., Chen, J., 2006. Elevational patterns of frog species richness and endemic richness in the Hengduan Mountains, China: geometric constraints, area and climate effects. Ecography 29, 919–927. https://doi.org/10.1111/j.2006.0906-7590.04802.x

Gao, D., Fu, L., Sun, J., Li, Y., Cao, Z., Liu, Y., Xu, P., Zhao, J., 2021. The mid-domain effect and habitat complexity applied to elevational gradients: Moss species richness in a temperate semihumid monsoon climate mountain of China. Ecology and Evolution 11, 7448–7460. https://doi.org/10.1002/ece3.7576

Geml, J., Morgado, L.N., Semenova-Nelsen, T.A., Schilthuizen, M., 2017. Changes in richness and community composition of ectomycorrhizal fungi among altitudinal vegetation types on Mount Kinabalu in Borneo. New Phytologist 215, 454–468. https://doi.org/10.1111/nph.14566

Heaney, L.R., 2001. Small mammal diversity along elevational gradients in the Philippines: an assessment of patterns and hypotheses. Global Ecology and Biogeography 10, 15–39. https://doi.org/10.1046/j.1466-822x.2001.00227.x

Herzog, S.K., Kessler, M., Bach, K., 2005. The elevational gradient in Andean bird species richness at the local scale: a foothill peak and a high-elevation plateau. Ecography 28, 209–222. https://doi.org/10.1111/j.0906-7590.2005.03935.x

Hu, Y., Jin, K., Huang, Z., Ding, Z., Liang, J., Pan, X., Hu, H., Jiang, Z., 2017. Elevational patterns of non-volant small mammal species richness in Gyirong Valley, Central Himalaya: Evaluating multiple spatial and environmental drivers. Journal of Biogeography 44, 2764–2777. https://doi.org/10.1111/jbi.13102

Kattan, G.H., Franco, P., 2004. Bird diversity along elevational gradients in the Andes of Colombia: area and mass effects. Global Ecology and Biogeography 13, 451–458. https://doi.org/10.1111/j.1466-822X.2004.00117.x

Kessler, M., 2001. Pteridophyte species richness in Andean forest in Bolivia. Biodiversity and Conservation 10, 1473–1495. https://doi.org/10.1023/A:1011811224595

Khatiwada, J.R., Zhao, T., Chen, Y., Wang, B., Xie, F., Cannatella, D.C., Jiang, J., 2019. Amphibian community structure along elevation gradients in eastern Nepal Himalaya. BMC Ecol 19, 19. https://doi.org/10.1186/s12898-019-0234-z

Kluge, J., Kessler, M., Dunn, R.R., 2006. What drives elevational patterns of diversity? A test of geometric constraints, climate and species pool effects for pteridophytes on an elevational gradient in Costa Rica. Global Ecology and Biogeography 15, 358–371. https://doi.org/10.1111/j.1466-822X.2006.00223.x

Lasmar, C.J., Rosa, C., Queiroz, A.C.M., Nunes, C.A., Imata, M.M.G., Alves, G.P., Nascimento, G.B., Ázara, L.N., Vieira, L., Louzada, J., Feitosa, R.M., Brescovit, A.D., Passamani, M., Ribas, C.R., 2021. Temperature and productivity distinctly affect the species richness of ectothermic and endothermic multitrophic guilds along a tropical elevational gradient. Oecologia 197, 243–257. https://doi.org/10.1007/s00442-021-05011-9

Lieberman, D., Lieberman, M., Peralta, R., Hartshorn, G., 1996. Tropical Forest Structure and Composition on a Large-Scale Altitudinal Gradient in Costa Rica. The Journal of Ecology 84. https://doi.org/10.2307/2261350

MALONZA, P.K., 2015. Patterns of reptile and amphibian species richness along elevational gradients in Mt. Kenya. Dongwuxue Yanjiu 36, 342–347. https://doi.org/10.13918/j.issn.2095-8137.2015.6.342

Mayr, A.V., Peters, M.K., Eardley, C.D., Renner, M.E., Röder, J., Steffan-Dewenter, I., 2020. Climate and food resources shape species richness and trophic interactions of cavity-nesting Hymenoptera. Journal of Biogeography 47, 854–865. https://doi.org/10.1111/jbi.13753

McCain, C.M., 2004. The mid-domain effect applied to elevational gradients: species richness of small mammals in Costa Rica. Journal of Biogeography 31, 19–31. https://doi.org/10.1046/j.0305-0270.2003.00992.x

Moura, M.R., Villalobos, F., Costa, G.C., Garcia, P.C.A., 2016. Disentangling the Role of Climate, Topography and Vegetation in Species Richness Gradients. PLoS One 11, e0152468. https://doi.org/10.1371/journal.pone.0152468

Musciano, M.D., Zannini, P., Ferrara, C., Spina, L., Nascimbene, J., Vetaas, O.R., Bhatta, K.P., d’Agostino, M., Peruzzi, L., Carta, A., Chiarucci, A., 2021. Investigating elevational gradients of species richness in a Mediterranean plant hotspot using a published flora. e50007. https://doi.org/10.21425/F5FBG50007

Nor, S.MD., 2001. Elevational diversity patterns of small mammals on Mount Kinabalu, Sabah, Malaysia. Global Ecology and Biogeography 10, 41–62. https://doi.org/10.1046/j.1466-822x.2001.00231.x

Rickart, E.A., 2001. Elevational diversity gradients, biogeography and the structure of montane mammal communities in the intermountain region of North America. Global Ecology and Biogeography 10, 77–100. https://doi.org/10.1046/j.1466-822x.2001.00223.x

Sam, K., Koane, B., Bardos, D.C., Jeppy, S., Novotny, V., 2019. Species richness of birds along a complete rain forest elevational gradient in the tropics: Habitat complexity and food resources matter. Journal of Biogeography 46, 279–290. https://doi.org/10.1111/jbi.13482

SÁnchez-Cordero, Ví., 2001. Elevation gradients of diversity for rodents and bats in Oaxaca, Mexico. Global Ecology and Biogeography 10, 63–76. https://doi.org/10.1046/j.1466-822x.2001.00235.x

Sanders, N.J., 2002. Elevational gradients in ant species richness: area, geometry, and Rapoport’s rule. Ecography 25, 25–32. https://doi.org/10.1034/j.1600-0587.2002.250104.x

Shuai, L.-Y., Ren, C.-L., Yan, W.-B., Song, Y.-L., Zeng, Z.-G., 2017. Different elevational patterns of rodent species richness between the southern and northern slopes of a mountain. Sci Rep 7, 8743. https://doi.org/10.1038/s41598-017-09274-2

Toledo-Garibaldi, M., Williams-Linera, G., 2014. Tree diversity patterns in successive vegetation types along an elevation gradient in the Mountains of Eastern Mexico. Ecological Research 29, 1097–1104. https://doi.org/10.1007/s11284-014-1196-4

Wu, Y., Colwell, R.K., Rahbek, C., Zhang, C., Quan, Q., Wang, C., Lei, F., 2013. Explaining the species richness of birds along a subtropical elevational gradient in the Hengduan Mountains. Journal of Biogeography 40, 2310–2323. https://doi.org/10.1111/jbi.12177
